# Development and Characterization of Cannabidiol Gummy Using 3D Printing

**DOI:** 10.3390/gels11030189

**Published:** 2025-03-08

**Authors:** Arvind Bagde, Mina Messiha, Mandip Singh

**Affiliations:** Pharmaceutical Sciences Department, Florida A&M University, Tallahassee, FL 32307, USA; arvind.bagde@famu.edu (A.B.); mina.messiha@famu.edu (M.M.)

**Keywords:** gummy, gel, gelatin, 3D printing, CBD, dysphagia

## Abstract

Oropharyngeal dysphagia and pain are prevalent concerns in the geriatric population. Therefore, this study investigates advances in the development of cannabidiol (CBD) gummies using 3D printing technology and compares them to commercially available molded gummies for pain management. A gelatin-based CBD formulation was prepared and printed using a syringe-based extrusion 3D printer. The formulation’s rheological properties were assessed, and the printed gummies were characterized using a texture analyzer. Drug content was analyzed using HPLC, and in vitro dissolution studies were conducted in phosphate buffer (pH 1.2 and 6.8). Our results demonstrated that the gelatin-based formulation had shear-thinning rheological properties for 3D printing at a temperature of 38.00 °C, filament diameter of 26 mm and flow of 110%. The optimized printing parameters produced gummies with higher elasticity compared to marketed gummies and comparable toughness. Drug content analysis showed 98.14 ± 1.56 and 97.97 ± 2.14% of CBD in 3D-printed and marketed gummies, respectively. Dissolution studies revealed that both gummy types released 100% of the drug within 30 min in both pH 1.2 and 6.8 buffers. Overall, 3D printing enables customizable CBD gummies with optimized release and offer a personalized and patient-friendly alternative to traditional oral forms for geriatric care.

## 1. Introduction

Swallowing difficulties, or dysphagia, are common among older adults due to age-related changes such as reduced muscle strength and coordination in the throat, as well as neurological conditions like strokes or Parkinson’s disease [1,2]. These issues can lead to serious health complications such as malnutrition, dehydration, and aspiration pneumonia [3,4,5]. Moreover, pain management in older adults is a complex issue influenced by multiple factors such as chronic conditions, reduced physiological reserves, and varying responses to medications [6]. Common sources of pain in the elderly include arthritis, neuropathies, fractures from falls, and post-surgical discomfort. Older adults may also experience pain related to age-related changes in joints and muscles, which can significantly impact their quality of life and functional abilities [7].

Modern medicine is increasingly focused on personalized treatments for each patient [8]. Gummy formulations, traditionally associated with confectionary treats, have gained recognition in healthcare for their distinctive attributes as oral dosage forms [9]. They are chewable, have pleasant flavors, and can improve patient compliance, especially for those who have difficulty swallowing pills [10,11,12]. As pharmaceutical medicine progresses, there has been a constant push for more customized therapy, tailored to a patient-specific level [13,14]. While medication doses can be adjusted with ease with parenteral therapy and liquid-based dosage forms for oral therapy, the dose customization options for solid oral dosage forms are far more limited [15]. Liquid dosage forms have inherent stability/degradation concerns and more stringent storage requirements compared to solid dosage forms [16]. Tablets of different premanufactured strengths may be combined or split in half, and capsule doses must be taken completely, as there is no way to measure portions of a dose accurately. Additionally, traditional solid dosage forms offer limited options for combining different medications, necessitating consuming multiple tablets or capsules or taking a combination tablet or capsule with predefined strengths of both medications. Several reports have extensively discussed the advantages of gummies in terms of patient adherence and acceptance [17,18,19]. It is reported that gummies provide a more user-friendly option compared to solid dosage forms. They reduce the risk of complications like esophageal impaction, a concern often associated with pills. Furthermore, gummies, due to their chewable nature and palatable flavors, have been instrumental in improving patient compliance [20,21,22].

In parallel, 3D printing technology has gained prominence in healthcare for its precision and adaptability in drug delivery systems [23,24]. The versatility of 3D printing allows for the fabrication of intricate structures with customized drug release profiles, thus paving the way for personalized medicine [25,26,27]. This capability is especially beneficial for populations requiring specific dosing regimens, such as pediatric or geriatric patients [28,29]. Furthermore, 3D printing facilitates the incorporation of various active pharmaceutical ingredients into a single dosage form, enabling complex drug release profiles and combination therapies. This approach not only enhances therapeutic efficacy but also improves patient adherence by reducing the pill burden [30,31]. Most published literature on 3D-printed medications focuses on tablets, with a few more recently published studies addressing alternative oral dosage forms [32,33,34,35]. As well, 3D-printed medicinal gummies with appealing designs and textures have been developed for children, offering a customizable and patient-friendly alternative to traditional solid dosage forms [36]. Furthermore, researchers have explored the use of 3D printing to fabricate lamotrigine-loaded gummies, offering a potentially more palatable and efficacious approach to pediatric anti-epileptic medication delivery [37]. Moreover, 3D-printed gummies have the potential to provide controlled drug release, enhancing therapeutic efficacy while minimizing side effects [38,39]. Using a syringe-based extrusion 3D printer, a gel formulation or “ink” with a prescribed concentration of active pharmaceutical ingredient (API) incorporated at a determined concentration, medicated gummies can be printed in mere minutes [36]. The stepper motors present in syringe-based extrusion printers allow for precise control over the amount of formulation extruded per unit of time [39]. Combined with a well-designed 3D model, the dose may be individualized by careful selection of the size and shape of the model printed [40,41]. This degree of dosage personalization, at the level of the individual patient, is impractical for traditional commercial large-scale drug manufacturers (mostly due to cost reasons), serving as an ideal niche for 3D printers to fill [30].

CBD (cannabidiol), a component of cannabis, has gained attention for its potential role in pain management among older adults. Studies have indicated that CBD’s anti-inflammatory and analgesic properties may help reduce pain intensity and improve overall quality of life without adverse effects [24,42,43,44,45]. Considering these therapeutic properties, the objective of this study was to develop 3D-printed CBD gummies for pain management in older adults and compare their release and mechanical properties with a marketed product. A commercially available CBD gummy was used as a reference due to its established role in the nutraceutical industry and widespread consumer use.

## 2. Results and Discussion

### 2.1. Formulation of CBD Gel and Its Rheological Studies

**Figure 1:**
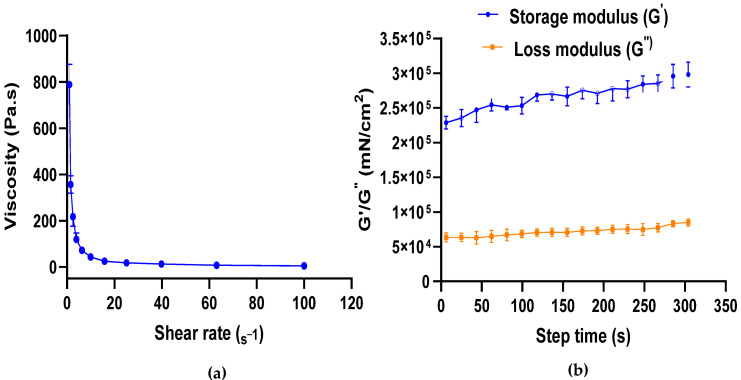
(**a**) Rheological study showing shear-thinning property of CBD gel at 38 °C (n = 3); (**b**) Rheological study of CBD gel showing higher storage than loss modulus at 38 °C (n = 3).

The CBD gel formulation with translucent appearance and yellowish color showed no sedimentation of particles. The results demonstrated a shear-thinning (pseudoplastic) behavior, where viscosity decreased from 788.53 ± 87.99 Pa·s to 4.36 ± 0.38 Pa·s with increasing shear rate. The storage modulus was found to be significantly higher (*p* < 0.001) than the loss modulus at 38 °C. The results also revealed that the tan delta value was below 1 (Figure 1).

### 2.2. 3D Printing of CBD Gummy and Characterization Using Texture Analyzer

**Figure 2:**
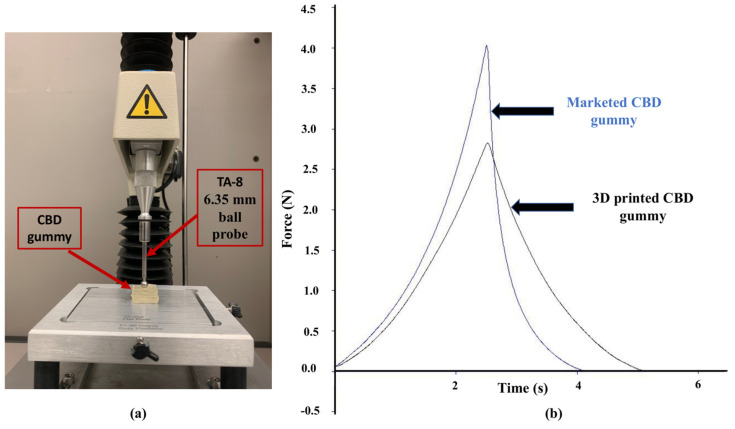
(**a**) Gummy characterization using a texture analyzer showing components of instrument, probe and gummy; (**b**) Texture analysis graphs showing firmness, toughness, elastic recovery, tackiness and resilience of 3D-printed and marketed molded gummies (n = 3).

Our results show that optimized printing parameters, including a printing temperature of 38 °C, a filament diameter of 26 mm and a flow of 110%, could successfully print a rectangular cuboid-shaped gummy with a dimension of 20 × 10 mm. Furthermore, results show no significant differences in gummies of the same size and shape, with less than 10% standard deviation, between gummies from the same batch (Supplementary Table S1). Texture analyzer data of the 3D-printed gummy showed firmness of 2.82 ± 0.15 N, toughness of 2.84 ± 0.18 N.s, tackiness of −0.0052 ± 0.0004 N, resilience of 91.00 ± 1.90% and elastic recovery of 98.58 ± 1.20%. Further, the marketed CBD gummy showed firmness of 4.03 ± 0.10 N, toughness of 3.51 ± 0.14 N.s, tackiness of −0.0033 ± 0.0003 N, resilience of 36.60 ± 3.60% and elastic recovery of 66.60 ± 0.40%. The 3D-printed gummy was found to have significantly higher (*p* < 0.01) elasticity with no significant difference (*p* = ns) in toughness (Figure 2 and Table 1). A drug content study showed 98.14 ± 1.56 and 97.97 ± 2.14% of CBD in the 3D-printed and marketed gummies, respectively.

### 2.3. In Vitro Dissolution Study

**Figure 3:**
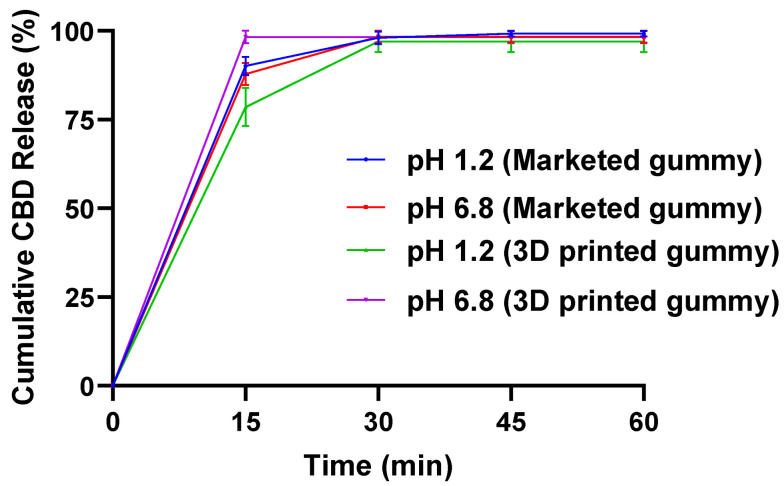
In vitro drug-release study showing complete drug release in 30 min in pH 1.2 and 6.8 buffer from both 3D-printed and marketed gummy (n = 3).

Our results show that the 3D-printed CBD gummy released 100% of the drug in < 1 h in both pH 1.2 and 6.8 buffers. Further, the marketed CBD gummy also showed a similar release pattern. It was also observed that both the 3D-printed and marketed gummies were dissolved completely within 30 min (Figure 3).

### 2.4. In Vitro Dissolution Study

Results show 98.17 ± 1.33 and 98.15 ± 0.84% of CBD after 15 and 30 days, respectively, at 4 °C. Moreover, gummies stored at room temperature showed 96.28 ± 0.87 and 92.35 ± 1.98% of CBD after 15 and 30 days, respectively. A texture analysis study showed no significant differences in firmness, toughness, tackiness, resilience and elastic recovery of CBD gummies at 15 and 30 days at room temperature. Gummies stored at 4 °C showed firmness of 4.17 ± 0.22 and 8.83 ± 0.31 N on days 15 and 30, respectively, toughness of 4.23 ± 0.12 and 8.98 ± 0.26 N.s on days 15 and 30, respectively, tackiness of −0.0051 ± 0.0003 and −0.0051 ± 0.0003 N, resilience of 89.13 ± 1.38 and 88.56 ± 1.86% and elastic recovery of 98.16 ± 1.31 and 98.10 ± 1.47% on days 15 and 30, respectively. The disintegration time for gummies stored at room temperature for 15 and 30 days was ~30 min. For gummies stored at 4 °C for 15 and 30 days, the disintegration time was ~45 min.

### 2.5. Discussion

The objective of this study was to develop 3D-printed CBD gummies for pain management by optimizing both formulation and printing parameters to achieve a product with mechanical property and release profiles similar to those of commercially available gummies. In this study, for the first time, we successfully printed CBD gummies, using a syringe-based extrusion 3D printer using gelation-based gel formulation, that demonstrated an immediate release of the drug and were comparable to marketed gummies.

In our preliminary studies, numerous batches were prepared to print gummies with various excipients, including xanthan gum, pregelatinized starch and carrageenan. However, the printed gummies lacked the desired dimensional integrity (shape and size). Our optimized formulation, containing very few excipients, and including gelatin and sugar, successfully printed CBD gummies with the desired shape and size. Gelatin, a natural polymeric peptide derived from the moderate hydrolysis of collagen, was selected due to its low cost, excellent gelling properties, solubility, biocompatibility, and emulsification capabilities, making it ideal for gummy manufacturing [46]. When dissolved in hot water and subsequently cooled, gelatin molecules form a network of bonds that trap water, resulting in a semi-solid, gel-like consistency. This process imparts the desired firmness and contributes to the elasticity and mouthfeel of the gummies [47]. Numerous studies have reported the use of gelatin in making gummies [48,49,50].

Our printing results show that CBD gummies with the desired size and shape were successfully printed at 38 °C. The use of a hot gelatin formulation in this study was essential for ensuring proper solubilization, homogeneity, and printability in extrusion-based 3D printing. Gelatin requires heating to fully dissolve in water, allowing for uniform hydration and dispersion of all components, including CBD and sugar, within the gummy matrix. Rheological studies at 38 °C showed that as the shear rate increased, the viscosity of the gel decreased significantly, suggesting the shear-thinning property of the gel [37]. The shear-thinning and thermo-gelling properties of gelatin are crucial for its use in 3D printing, as shear-thinning facilitates smooth extrusion from the syringe-based printer, while thermo-gelling ensures rapid solidification after deposition. It is well known that temperature significantly affects the viscosity of gel formulation, primarily due to the presence of gelatin [51]. Le et al. also investigated the influence of temperature on the viscosity of gels containing gelatin, low-acyl gellan, and various sweeteners (maltol, erythritol, sorbitol, and xylitol). Their findings confirmed a relationship between these two factors, reporting a gelation temperature of 40.59 °C for their specific gel formulation [52]. CBD gel formulation showed a higher storage modulus than loss modulus with a tan delta value below 1, suggesting the elastic nature of the gel. A tan delta value below 1 generally indicates that the material exhibits more elastic (solid-like) behavior, whereas a value greater than 1 suggests a more viscous (liquid-like) state. In our study, a tan δ below 0.5 confirms that the storage modulus (G′) dominates over the loss modulus (G″), indicating that the material behaves predominantly as a solid-like viscoelastic gel rather than a purely liquid phase. However, it does not imply a completely rigid solid state, as it still retains some degree of viscoelasticity essential for chewability and flexibility in gummy formulations [53]. Tagami et al. emphasized that adjusting the viscosity of the gel formulation is crucial to achieving good printability when utilizing an extrusion-type 3D printer to print gummies. An extremely low viscosity makes it difficult to laminate gel and causes it to flow from the nozzle with ease. An extremely high-viscosity gel, on the other hand, results in a semi-solid product that cannot be extruded out the nozzle without significant pressure [37]. Gummies were printed at a print bed temperature of 21–25 °C (room temperature) to facilitate controlled gelation without premature solidification during the extrusion process. A higher print bed temperature could delay gelation and affect the final mechanical properties of the printed gummies. At higher temperatures, the gelatin-based formulation may soften due to its thermosensitive nature. To mitigate this, formulations could be optimized by incorporating additional stabilizing agents, such as carrageenan or hydrocolloids, which improve heat resistance [46].

Textural analysis demonstrated that 3D-printed CBD gummies exhibited firmness and toughness comparable to commercially available molded CBD gummies. This suggests favorable consumer perception in terms of chewability [54]. Kean et al., who developed an isoniazid gummy (containing gelatin, polyvinyl pyrrolidone, and magnesium stearate), also characterized gummies using a texture analyzer. Their study revealed that the gummy exhibited a hardness of 37.260 ± 4.66 N and a resilience of 0.542 ± 0.029 [55]. In comparison to their study, our 3D-printed CBD gummy had a hardness of 2.82 ± 0.15 N, which was comparable to the marketed CBD gummy (hardness of 4.03 ± 0.10 N). Furthermore, the 3D-printed gummies showed superior elasticity compared to the marketed gummies. This enhanced elasticity can be attributed to the viscoelastic properties of gelatin, a crucial component of the formulation. Gelatin’s unique property allows it to undergo deformation under stress but readily recover its original shape upon relaxation. This characteristic may contribute to a more pleasurable mouthfeel for the consumer [56].

An in vitro release study revealed no statistically significant differences in the dissolution profiles between 3D-printed and commercially available CBD gummies. Both formulations exhibited complete drug release within one hour in both acidic and basic pH buffers. This rapid dissolution rate in the simulated gastric fluid suggests that the gummies would disintegrate quickly in the mouth, a desirable characteristic for any gummy product. Our results are in concordance with Tagami et al., who studied 3D printing of gummy drug formulations composed of gelatin and an HPMC-based hydrogel and showed rapid release of lamotrigine from the gummies in in vitro dissolution studies [37]. In another study, researchers designed a polymeric gummy drug formulation (P-GDF) containing a first-line antitubercular agent, isoniazid, using a combined solid–liquid dispersion and temperature dependent sol–gel processing technique and showed immediate release of drug from the gummies in in vitro dissolution studies in both acidic and basic pH buffers [55]. The stability study showed no significant change in drug content in the gummies stored at 4 °C, indicating that CBD-containing gummies remain stable at this temperature over a 30-day period. However, when stored at room temperature, approximately 8% of the CBD degraded by the end of the 30 days. These findings are consistent with studies reported by other researchers. Mazzetti et al. reported that the stability of CBD is influenced by several factors, with temperature being particularly critical. While CBD is highly unstable at room temperature, it remains stable for at least 12 months when stored at 5 °C [57]. A study by Kosović et al. found that CBD in solid powder form remained mostly stable over one year. In their study, 5 mg of marketed CBD, both in solid powder form and as an oil solution, were exposed for 7, 14, 30, 60, 90, 180, 270, and 365 days to precisely controlled temperature and humidity conditions (25 °C ± 2 °C/60% RH ± 5% and 40 °C ± 2 °C/75% RH ± 5%) in both open and closed vials kept in the dark. However, the CBD oil was much more susceptible to degradation due to temperature, humidity, and air exposure. Significant degradation occurred between 90 and 180 days in open vials at 40 °C/75% RH, with complete degradation observed after approximately 270 days. Artificial light exposure did not significantly affect CBD stability [58]. Texture analysis revealed no significant differences in firmness, toughness, tackiness, resilience, and elastic recovery of CBD gummies at room temperature. This indicates that room temperature (25 °C ± 2 °C) does not impact the firmness or other physical properties of the gummies. However, gummies stored at 4 °C exhibited a significant increase in firmness and toughness, while their elasticity remained unchanged. The gummy disintegration study showed no significant differences in disintegration time between gummies stored at room temperature and those stored at 4 °C. This suggests that while the gummies became harder at 4 °C, their disintegration time was not impacted, as they softened at 37 °C and melted in the buffer.

Future investigations will focus on a systematic approach to optimizing ingredient ratios and mechanical properties to enhance the formulation’s stability and performance. Additionally, research efforts will be directed toward the development of multilayered gummies incorporating multiple active pharmaceutical ingredients (APIs) to facilitate personalized therapeutic strategies. This approach may improve patient adherence and therapeutic efficacy by enabling the co-administration of multiple agents within a single dosage form. Further studies will also aim to explore a range of cannabidiol (CBD) concentrations to establish a comprehensive understanding of its pharmacokinetic and pharmacodynamic profiles at varying dosages. This will involve evaluating the stability, bioavailability, and therapeutic outcomes of different formulations to optimize dosing strategies for individualized treatment regimens. Such investigations will contribute to refining precision medicine approaches for CBD-based therapies, ensuring consistent efficacy and safety across diverse patient populations. Moreover, while this study provides foundational insights, additional research is required to elucidate the stability and degradation kinetics of CBD under different environmental conditions. Future studies will focus on characterizing potential degradation pathways and assessing the impact of critical external factors, including temperature, humidity, and light exposure, on CBD stability. Overall, this study successfully developed 3D-printed CBD gummies for pain management in older adults with optimized mechanical properties and immediate drug release comparable to commercial products by using gelatin and sugar. The printed gummies exhibited superior elasticity due to the viscoelastic properties of gelatin.

## 3. Conclusions

This study successfully demonstrated the potential of 3D printing technology to develop customized CBD gummy formulations with required mechanical and drug release properties for pain management in geriatric patients. By optimizing the formulation with key excipients like gelatin and sugar, the printed gummies achieved the desired shape, size, and immediate drug release comparable to commercially available products. The study also highlights the critical influence of temperature and viscosity on the printability and structural integrity of gelatin-based gels, emphasizing the importance of precise parameter control in the 3D printing process. This research underscores the versatility and efficacy of 3D printing in pharmaceutical manufacturing, paving the way for innovative, patient-centric drug delivery systems.

## 4. Materials and Methods

### 4.1. Materials

The cannabidiol (CBD) used in this study was a CBD super isolate obtained from Open Book Extracts (Roxboro, NC, USA), as confirmed by the Certificate of Analysis (COA) from ACS Laboratory (Sun City Center, FL, USA) (COA is attached in the Supplementary File). The batch used (Batch # BCA-000859-230719) was extracted from hemp and was independently tested for potency, heavy metals, mycotoxins, pesticides, residual solvents, and pathogenic microbiology, passing all compliance tests. The isolate was found to be of exceptionally high purity (99.955% *w*/*w* active CBD) with no detectable Δ9-THC, THCA, CBG, CBN, or other cannabinoids above the limit of quantitation (LOQ). Superclear gelatin, transparent edible gelat (unflavoured gelatin powder), was obtained from Custom Collagen, Inc, Addison, IL, USA. Granulated sugar was purchased from Walmart, Tallahassee, FL, USA. Macrogolglycerol ricinoleate (polyoxyl 35 castor oil) was gifted by BASF corporation, Florham Park, NJ, USA. Ethanol was purchased from Sigma Aldrich, St. Louis, MO, USA. A Foodbot 3D printer was purchased from CHANGXING SHYIN TECH Co., Ltd., Hangzhou, China. A texture analyzer (TA.XTPlus) and a TA-8 (6.35 mm) probe were purchased from Texture Technologies Corp. and Stable Micro Systems, Ltd., Hamilton, MA, USA. A rheometer was purchased from TA instruments, New Castle, DE, USA (Model: Discovery Hybrid Rheometer (DHR) 20). CBD gummies were obtained from Midwest Hemp Authority, Deerfield, IL, USA to study as a comparison to 3D-printed gummies. The selection of the reference commercial product was based on its availability and relevance to the study, ensuring a meaningful comparison with the 3D-printed gummies. We do not have any financial relationships with the company that provided the commercial gummies; the samples were provided free of charge for research purposes to our academic institution, without any obligation or influence on the study outcomes. Furthermore, this research is conducted with an academic and scientific approach, and we are not endorsing or promoting any specific commercial product. The inclusion of a commercially available gummy was solely for the purpose of evaluating mechanical properties, drug content, and dissolution characteristics in comparison to the 3D-printed formulation.

### 4.2. Methods

#### 4.2.1. Formulation of CBD Gel

To ensure uniform dispersion and solubility of CBD within the gel formulation, CBD solubilization was performed in a separate Eppendorf tube before incorporation into the main gel matrix. The CBD isolate (0.56% *w*/*v*; 99.955% purity, as confirmed by COA) was first dissolved in 10% *w*/*v* ethanol. Following this, 5% *w*/*v* polyoxyl 35 castor oil (macrogolglycerol ricinoleate) was added to further enhance the solubility of CBD in the solution. This pre-dissolution step was necessary due to the hydrophobic nature of CBD, which limits its direct solubility in aqueous solutions. The solution was vortexed for 1 min at room temperature to ensure complete solubilization of CBD, preventing precipitation and promoting uniform dispersion, as described in our previous study [59]. In a separate beaker, 31.25% *w*/*v* of granulated sugar was dissolved in preheated deionized water at 80 °C under continuous stirring at 500 rpm. The heating process facilitated the complete solubilization of sugar, ensuring a clear and uniform solution before the addition of other components. Once fully dissolved, the pre-solubilized CBD solution was gradually introduced into the sugar solution while stirring at 500 rpm for an additional 5 min. Following the incorporation of CBD, 18.75% *w*/*v* of gelatin was gradually added to the mixture while maintaining the temperature at 80 °C. Stirring speed was 500 rpm, and mixing was continued for 15–20 min to allow for the complete hydration and dissolution of gelatin. This step ensured the formation of a homogeneous gel matrix for 3D printing. Once the formulation was fully homogenized, it was transferred into a printing syringe and allowed to cool to 38 °C, the optimal temperature identified for maintaining the gel’s extrusion properties and structural integrity. Controlled cooling prevented premature solidification while ensuring that the gel remained extrudable and printable. The 3D printing process was carried out at 38 °C, ensuring precise layer deposition, uniform gummy dimensions, and structural stability. This optimized formulation and process allowed for the production of customizable CBD gummies with homogeneous drug distribution and reproducible mechanical properties comparable to those of commercially available products.

#### 4.2.2. Rheological Study of CBD Gel

The rheological properties of the CBD gel were assessed using a DHR 20 rheometer (TA Instruments) equipped with a plate–plate geometry (25 mm diameter). The flow behavior and viscoelastic properties of the formulation were evaluated using steady-shear and oscillatory rheological tests to determine its suitability for extrusion-based 3D printing. For each measurement, about 300 mg sample was used with a measuring gap of 0.5 mm. Briefly, for the flow sweep experiment, the formulation was run at 38 °C, soak time of 180 s, and shear rate of 1.0 s^−1^ to 100.0 s^−1^. Data were then collected and plotted as viscosity (Pa.s) vs. shear rate (s^−1^). For the oscillation time sweep experiment, the formulation was run at 38 °C, soak time of 180 s, strain of 1% and angular frequency of 10 rad/s. Further, the data were plotted as Storage, G′ and Loss modulus, G′′ (mN/cm^2^) vs. step time, ts (s) graph format [37,53,60].

#### 4.2.3. Three-Dimensional Printing of CBD Gummy

**Figure 4:**
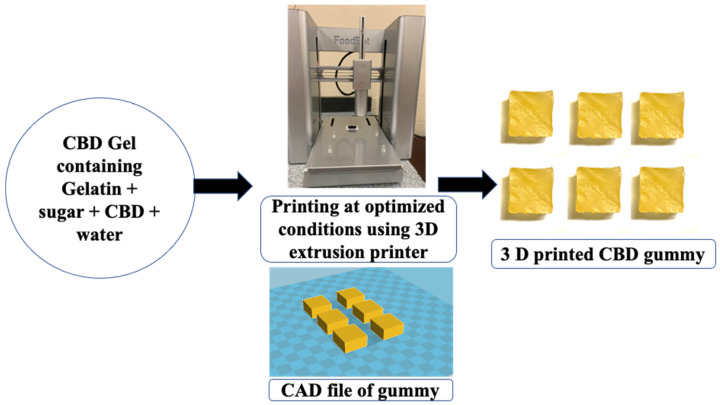
Illustration showing steps involved in printing CBD gummy using 3D printing.

A computer-aided design (CAD) file of a 20 × 10 mm rectangular cuboid-shaped gummy was created using Cura 15.02.1 software and imported to the 3D printer as a STL file. Six gummies were printed at once, with a layer height of 0.34 mm, shell thickness of 0.8 mm, bottom/top thickness of 1.2 mm, fill density of 100%, print speed of 20 mm/s, printing temperature of 38 °C, filament diameter of 26 mm and flow of 110%. The print bed temperature was not controlled. Gummies were printed at room temperature (21–25 °C) to facilitate controlled gelation without premature solidification during the extrusion process (Figure 4). Furthermore, gummies were also printed in different shapes, including cube, cylinder, and ellipsoid, with 10 mm each side, 10 × 4 × 10 mm and 10 × 10 mm sizes, respectively, with the optimized printing settings (Supplementary Figure S1). In the Foodbot 3D printer (CHANGXING SHYIN TECH Co., Ltd., Zhejiang province, China), the filament diameter setting refers to the diameter of the extruded material (gel formulation) as it exits the nozzle during semisolid extrusion-based 3D printing.

#### 4.2.4. Characterization of the Gummy Using Texture Analyzer

For 3D printed CBD gummies, a TA-8 1⁄4” ball probe was used for characterization of the gummy using a texture analyzer. The test was performed in return to start in compression mode with 1.0 mm/s Pre-Test Speed, 2.0 mm/s Test Speed, 2.0 mm/s Post-Test Speed, 5.0 mm Target Distance and 0.049 N Automatic Trigger. Firmness, toughness, tackiness, resilience and elastic recovery were then calculated using the data obtained from the texture analyzer. As a comparison, CBD gummies which were made by the traditional molding method were also used as comparison and were gifted by Midwest Hemp Authority, Chicago, IL, USA [37,48].

#### 4.2.5. HPLC Analysis

HPLC analysis was conducted with a Waters e2695 separation module and a Waters 2998 photodiode array detector (PDA) (Waters Technology Corporation, Milford, MA, USA). A reverse-phase C18 column (Nova-Pak^®^ 3.5 µm, 3.9 × 150 mm; Waters Technology Corporation, USA) with a guard column (Symmetry^®^, reverse-phase, C18) was used for the elution of samples. A stock solution of CBD (1 mg/mL) was prepared in methanol. Serial dilutions were then prepared at 0.78, 1.56, 3.125, 6.25, 12.5, 25, 50, and 100 µg/mL using a methanol/phosphate buffer mixture (pH 1.2/6.8) (50:50). A mobile phase containing 85% methanol and 15% water was used at a flow rate of 0.5 mL/min with an injection volume of 20 µL. The retention time was found to be 4.44 min. The calibration curve (peak area vs. concentration) was generated over the range of 0.78–100 µg/mL and was found to be linear with correlation coefficients of 0.986 and 0.99 for the methanol/phosphate buffer mixture (pH 6.8) (50:50) and the methanol/phosphate buffer mixture (pH 1.2) (50:50), respectively [45,61].

#### 4.2.6. Drug Content Study

Gummies were analyzed for the drug content using HPLC. Briefly, a CBD gummy was dissolved in water at 37 °C with constant stirring for 2 h. Samples of 1 mL were then taken from the solution and diluted in a 1:1 ratio with methanol. Finally, the samples were centrifuged at 14,000 RPM to discard any particles. Supernatant was then collected and injected in HPLC for the drug content analysis [23,24,55]. In our previously published study [59], we extensively investigated the stability of CBD at pH 1.2 (simulated gastric fluid) and pH 6.8 (simulated intestinal fluid). Our findings demonstrate that CBD remains stable under these conditions.

#### 4.2.7. In Vitro Dissolution Study

An in vitro dissolution study was carried out using USP dissolution apparatus Type II with a small-volume adaptor assembly in phosphate buffers (pH 1.2 and 6.8). Briefly, dissolution medium (50 mL) was allowed to equilibrate to 37 °C for about 45 min before adding the gummies to the flasks. After the equilibration, a gummy was added to each flask, and the study was started at 100 rpm. Samples (0.5 mL) were then collected at 15, 30, 45, 60, 75, 90, 105 and 120 min for analysis using HPLC, and fresh 0.5 buffer was added to the flask. The release study samples were diluted with the mobile phase (in a ratio of 50:50) [37,55].

#### 4.2.8. Stability Study

CBD gummies were kept in a sealed airtight aluminum envelope at room temperature (25 °C ± 2 °C) and at 2–5 °C in the fridge. The gummies were then analyzed for their drug content, texture analysis and disintegration time on days 15 and 30. Disintegration time for the gummies was evaluated in a USP dissolution apparatus Type II with a small-volume adaptor assembly in phosphate buffers (pH 6.8). Briefly, dissolution medium (50 mL) was allowed to equilibrate to 37 °C for about 45 min before adding the gummies to the flasks. After the equilibration, a gummy was added to each flask and the study was started at 100 rpm. The time at which the gummies fully dissolved in the buffer was recorded.

#### 4.2.9. Statistical Analysis

Based on at least three repetitions, the raw data findings were displayed as the mean ± standard deviation (SD). The t-test with unpaired experimental design with Welch’s correction was used for the statistical analysis of the data. Differences between groups were considered statistically significant at *p* < 0.05, while *p* = ns indicated no significant difference. Statistical analyses were conducted using GraphPad Prism 5.0 (GraphPad Software, Inc., San Diego, CA, USA).

## Figures and Tables

**Table 1:** Gummy characterization using texture analyzer showing firmness, toughness, elastic recovery, tackiness and resilience of 3D-printed and marketed molded gummies (n = 3).

Gummy	Firmness (N)	Toughness (N.s)	Resilience (%)	Elastic Recovery (%)	Tackiness (N)
3D-printed	2.82 ± 0.15	2.84 ± 0.18	91.00 ± 1.90	98.58 ± 1.20	−0.0052 ± 0.0004
Marketed	4.03 ± 0.10	3.51 ± 0.14	36.60 ± 3.60	66.60 ± 0.40	−0.0033 ± 0.0003

## Data Availability

The original contributions presented in this study are included in the article/Supplementary Material. Further inquiries can be directed to the corresponding author.

## Supplementary Materials

The following supporting information can be downloaded at https://www.mdpi.com/article/10.3390/gels11030189/s1, Figure S1: CBD gummies with different shapes and sizes printed using 3D printer, and Table S1: Different batches of gummies with various shapes and sizes showing no significant differences in their weight, size, and shape.

## Abbreviations

The following abbreviations are used in this manuscript:

CBD	Cannabidiol
HPLC	High-pressure liquid chromatography
P-GDF	Polymeric gummy drug formulation
HPMC	Hydroxy propyl methyl cellulose
