# Supplementary material for: Development and Characterization of Cannabidiol Gummy Using 3D Printing
Source: Gels. 2025 Mar 8;11(3):189. doi: 10.3390/gels11030189 (PMC11941846; doi:10.3390/gels11030189)
Supplement: Supplementary file 1 [file gels-11-00189-s001.zip › gels-3445528-Certificate of Analysis .pdf]

ACS LABORATORY | CANNABIS & HEMP BEYOND COMPLIANCE
721 Cortaro Dr.
Sun City Center, FL 33573
www.acslabcannabis.com
DEA No. RA0571996
FL License # CMTL-0003
CLIA No. 10D1094068

TESTED SAFE CERTIFIED HEMP EXTRACT

CBD Super Isolate - Finished Bulk - ITM000016
Sample Matrix: CBD/HEMP Derivative Products (Ingestion)

Certificate of Analysis

Compliance Test

Client Information:

OPEN BOOK EXTRACTS
317 LUCY GARRETT RD
ROXBORO, NC 27574

Batch # BCA-000859-230719
Batch Date: 2023-08-07
Extracted From: Hemp

Test Reg State: Florida

Order # OPE230807-010001
Order Date: 2023-08-07
Sample # AAET197

Sampling Date: 2023-08-08
Lab Batch Date: 2023-08-08
Completion Date: 2023-08-13

Initial Gross Weight: 113.987 g

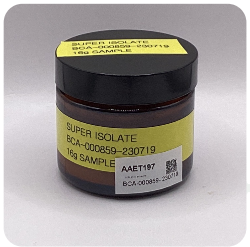

Product Image

Test	Status
Potency	Tested
Heavy Metals	Passed
Mycotoxins	Passed
Pesticides	Passed
Residual Solvents	Passed
Pathogenic Microbiology	Passed

Potency 10

Tested

Specimen Weight: 101.190 mg

SOP13.001 (LCUV)

Analyte	Dilution (1:n)	LOD (%)	LOQ (%)	Result (mg/g)	(%)
CBD	10.000	5.40E-5	0.015	999.550	99.955
CBDV	10.000	6.50E-5	0.015	0.350	0.035
CBC	10.000	1.80E-5	0.015	<LOQ	<LOQ
CBDA	10.000	1.00E-5	0.015	<LOQ	<LOQ
CBG	10.000	2.48E-4	0.015	<LOQ	<LOQ
CBGA	10.000	8.00E-5	0.015	<LOQ	<LOQ
CBN	10.000	1.40E-5	0.015	<LOQ	<LOQ
Delta-9 THC	10.000	1.30E-5	0.015	<LOQ	<LOQ
THCA-A	10.000	3.20E-5	0.015	<LOQ	<LOQ
THCV	10.000	7.00E-6	0.015	<LOQ	<LOQ
Total Active CBD	10.000			999.550	99.955
Total Active THC	10.000			<LOQ	<LOQ

Potency Summary

**Total Active THC** - None Detected	**Total Active CBD** 99.955%
**Total CBG** - None Detected	**Total CBN** - None Detected
**Other Cannabinoids** 0.035%	**Total Cannabinoids** 99.990%

Aixia Sun — Lab Director/Principal Scientist
D.H.Sc., M.Sc., B.Sc., MT (AAB)

A2LA ACCREDITED CERT #6786.01 · AHCA · DEA REGISTERED LABORATORY #RA0571996

Definitions and Abbreviations used in this report: Total Active CBD = CBD + (CBD-A * 0.877), *Total CBDV = CBDV + (CBDVA * 0.87), Total Active THC = THCA-A * 0.877 + Delta 9 THC, Total THCV = THCV + (THCVA * 0.87) , CBG Total = (CBGA * 0.877) + CBG, CBN Total = (CBNA * 0.877) + CBN, Total CBC = CBC + (CBCA * 0.877), Total THC-O-Acetate = Delta 8 THC-O-Acetate + Delta 9 THC-O-Acetate, Total THCP = Delta8-THCP + Delta9-THCP, Other Cannabinoids Total = Total Cannabinoids - All the listed cannabinoids on the summary section, Total Detected Cannabinoids = Delta6a10a-THC + Delta8-THC + Total CBN + CBT + CBE + Delta8-THCV + Total CBG + Total CBD + Total THCV + CBL + Total THC + Total CBC + Total CBDV + Delta10-THC + Total THC-O-Acetate + Total THCP. (mg/ml) = Milligrams per Milliliter, LOQ = Limit of Quantitation, LOD = Limit of Detection, Dilution = Dilution Factor (ppb) = Parts per Billion, (%) = Percent, (cfu/g) = Colony Forming Unit per Gram (cfu/g) = Colony Forming Unit per Gram, , LOD = Limit of Detection, (µg/g) = Microgram per Gram (ppm) = Parts per Million, (ppm) = (µg/g), (aw) = Water Activity, (mg/Kg) = Milligram per Kilogram, ACS uses simple acceptance criteria. Passed – Analyte/microbe is not detected or is at the level below the action limit per FL rule 64ER20-39, 5K-4.036, 5K-4.034. Failed – Analyte/microbe is at the level that equal or above the action limit per FL rule 64ER20-39, 5K-4.036, 5K-4.034 Sample not received via laboratory sampling.

This report shall not be reproduced, without written approval, from ACS Laboratory. The results of this report relate only to the material or product analyzed. Test results are confidential unless explicitly waived otherwise. ACS Laboratory is accredited to the ISO/IEC 17025:2017 Standard.

QA By: 1042 on 2023-08-13 14:04:39 V1

ACS LABORATORY | CANNABIS & HEMP BEYOND COMPLIANCE
721 Cortaro Dr.
Sun City Center, FL 33573
www.acslabcannabis.com
DEA No. RA0571996
FL License # CMTL-0003
CLIA No. 10D1094068

TESTED SAFE CERTIFIED HEMP EXTRACT

CBD Super Isolate - Finished Bulk - ITM000016
Sample Matrix: CBD/HEMP Derivative Products (Ingestion)

Certificate of Analysis

Compliance Test

Client Information:
OPEN BOOK EXTRACTS
317 LUCY GARRETT RD
ROXBORO, NC 27574

Batch # BCA-000859-230719
Batch Date: 2023-08-07
Extracted From: Hemp

Test Reg State: Florida

Order # OPE230807-010001
Order Date: 2023-08-07
Sample # AAET197

Sampling Date: 2023-08-08
Lab Batch Date: 2023-08-08
Completion Date: 2023-08-13

Initial Gross Weight: 113.987 g

Pathogenic Microbiology - SE (MicroArray)

Specimen Weight: 1028.600 mg

Passed
SOP13.019 (Micro Array)

Dilution Factor: 1.000

Analyte	Result (cfu/g)	Analyte	Result (cfu/g)
Salmonella	Absence in 1g	STEC E. Coli	Absence in 1g

Aixia Sun Lab Director/Principal Scientist
D.H.Sc., M.Sc., B.Sc., MT (AAB)

Definitions are found on page 1

This report shall not be reproduced, without written approval, from ACS Laboratory. The results of this report relate only to the material or product analyzed. Test results are confidential unless explicitly waived otherwise. ACS Laboratory is accredited to the ISO/IEC 17025:2017 Standard.

ACCREDITED CERT #6786.01

DEA REGISTERED LABORATORY #RA0571996

AHCA

ACS LABORATORY | CANNABIS & HEMP BEYOND COMPLIANCE
721 Cortaro Dr.
Sun City Center, FL 33573
www.acslabcannabis.com
DEA No. RA0571996
FL License # CMTL-0003
CLIA No. 10D1094068

TESTED SAFE CERTIFIED HEMP EXTRACT

CBD Super Isolate - Finished Bulk - ITM000016
Sample Matrix: CBD/HEMP Derivative Products (Ingestion)

Certificate of Analysis
Compliance Test

Client Information:
OPEN BOOK EXTRACTS
317 LUCY GARRETT RD
ROXBORO, NC 27574

Batch # BCA-000859-230719
Batch Date: 2023-08-07
Extracted From: Hemp

Test Reg State: Florida

Order # OPE230807-010001
Order Date: 2023-08-07
Sample # AAET197

Sampling Date: 2023-08-08
Lab Batch Date: 2023-08-08
Completion Date: 2023-08-13

Initial Gross Weight: 113.987 g

Heavy Metals
Specimen Weight: 250.100 mg

Passed
SOP13.048 (ICP-MS)

Dilution Factor: 199

Analyte	LOD (ppb)	LOQ (ppb)	Action Level (ppb)	Result (ppb)	Analyte	LOD (ppb)	LOQ (ppb)	Action Level (ppb)	Result (ppb)
Arsenic (As)	4.83	100	1500	<LOQ	Lead (Pb)	11.76	100	500	<LOQ
Cadmium (Cd)	.64	100	500	<LOQ	Mercury (Hg)	.58	100	3000	<LOQ

Mycotoxins
Specimen Weight: 601.900 mg

Passed
SOP13.007 (LCMS)

Dilution Factor: 2.490

Analyte	LOD (ppb)	LOQ (ppb)	Action Level (ppb)	Result (ppb)	Analyte	LOD (ppb)	LOQ (ppb)	Action Level (ppb)	Result (ppb)
Aflatoxin B1	3.0400E-1	6	20	<LOQ	Aflatoxin G2	2.7100E-1	6	20	<LOQ
Aflatoxin B2	7.7000E-2	6	20	<LOQ	Ochratoxin A	7.5400E-1	3.8	20	<LOQ
Aflatoxin G1	3.0400E-1	6	20	<LOQ					

Residual Solvents - FL (CBD)
Specimen Weight: 302.500 mg

Passed
SOP13.039 (GCMS)

Dilution Factor: 50.000

Analyte	LOD (ppm)	LOQ (ppm)	Action Level (ppm)	Result (ppm)	Analyte	LOD (ppm)	LOQ (ppm)	Action Level (ppm)	Result (ppm)
1,1-Dichloroethene	0.0094	0.16	8	<LOQ	Heptane	0.0013	1.39	5000	51.350
1,2-Dichloroethane	0.0003	0.04	5	<LOQ	Hexane	0.068	1.17	290	<LOQ
Acetone	0.015	2.08	5000	<LOQ	Isopropyl alcohol	0.0048	1.39	500	<LOQ
Acetonitrile	0.06	1.17	410	<LOQ	Methanol	0.0005	0.69	3000	<LOQ
Benzene	0.0002	0.02	2	<LOQ	Methylene chloride	0.0029	2.43	600	<LOQ
Butanes	0.4167	2.5	2000	<LOQ	Pentane	0.037	2.08	5000	<LOQ
Chloroform	0.0001	0.04	60	<LOQ	Propane	0.031	5.83	2100	<LOQ
Ethanol	0.0021	2.78	5000	<LOQ	Toluene	0.0009	2.92	890	<LOQ
Ethyl Acetate	0.0012	1.11	5000	<LOQ	Total Xylenes	0.0001	2.92	2170	<LOQ
Ethyl Ether	0.0049	1.39	5000	<LOQ	Trichloroethylene	0.0014	0.49	80	<LOQ
Ethylene Oxide	0.0038	0.1	5	<LOQ					

Aixia Sun Lab Director/Principal Scientist
D.H.Sc., M.Sc., B.Sc., MT (AAB)

Definitions are found on page 1
This report shall not be reproduced, without written approval, from ACS Laboratory. The results of this report relate only to the material or product analyzed. Test results are confidential unless explicitly waived otherwise. ACS Laboratory is accredited to the ISO/IEC 17025:2017 Standard.

ACCREDITED CERT #6786.01
DEA REGISTERED LABORATORY #RA0571996
AHCA

QA By: 1042 on 2023-08-13 14:04:39 V1

ACS LABORATORY | CANNABIS & HEMP BEYOND COMPLIANCE

721 Cortaro Dr.
Sun City Center, FL 33573
www.acslabcannabis.com
DEA No. RA0571996
FL License # CMTL-0003
CLIA No. 10D1094068

TESTED SAFE CERTIFIED HEMP EXTRACT

CBD Super Isolate - Finished Bulk - ITM000016
Sample Matrix: CBD/HEMP Derivative Products (Ingestion)

Certificate of Analysis

Compliance Test

Client Information:

OPEN BOOK EXTRACTS
317 LUCY GARRETT RD
ROXBORO, NC 27574

Batch # BCA-000859-230719
Batch Date: 2023-08-07
Extracted From: Hemp

Test Reg State: Florida

Order # OPE230807-010001
Order Date: 2023-08-07
Sample # AAET197

Sampling Date: 2023-08-08
Lab Batch Date: 2023-08-08
Completion Date: 2023-08-13

Initial Gross Weight: 113.987 g

Pesticides

Passed

Specimen Weight: 601.900 mg

SOP13.007 (LCMS/GCMS)

Dilution Factor: 2.490

Analyte	LOD (ppb)	LOQ (ppb)	Action Level (ppb)	Result (ppb)	Analyte	LOD (ppb)	LOQ (ppb)	Action Level (ppb)	Result (ppb)
Abamectin	2.8800E-1	28.23	300	<LOQ	Fludioxonil	1.7400E+0	48	3000	<LOQ
Acephate	2.3000E-2	30	3000	<LOQ	Hexythiazox	4.9000E-2	30	2000	<LOQ
Acequinocyl	9.5640E+0	48	2000	<LOQ	Imazalil	2.4800E-1	30	100	<LOQ
Acetamiprid	5.2000E-2	30	3000	<LOQ	Imidacloprid	9.4000E-2	30	3000	<LOQ
Aldicarb	2.6000E-2	30	100	<LOQ	Kresoxim Methyl	4.2000E-2	30	1000	<LOQ
Azoxystrobin	8.1000E-2	10	3000	<LOQ	Malathion	8.2000E-2	30	2000	<LOQ
Bifenazate	1.4150E+0	30	3000	<LOQ	Metalaxyl	8.1000E-2	10	3000	<LOQ
Bifenthrin	4.3000E-2	30	500	<LOQ	Methiocarb	3.2000E-2	30	100	<LOQ
Boscalid	5.5000E-2	10	3000	<LOQ	Methomyl	2.2000E-2	30	100	<LOQ
Captan	6.1200E+0	30	3000	<LOQ	methyl-Parathion	1.7100E+0	10	100	<LOQ
Carbaryl	2.2000E-2	10	500	<LOQ	Mevinphos	2.1500E+0	10	100	<LOQ
Carbofuran	3.4000E-2	10	100	<LOQ	Myclobutanil	1.0290E+0	30	3000	<LOQ
Chlorantraniliprole	3.3000E-2	10	3000	<LOQ	Naled	9.5000E-2	30	500	<LOQ
Chlordane	1.0000E+1	10	100	<LOQ	Oxamyl	2.5000E-2	30	500	<LOQ
Chlorfenapyr	3.4000E-2	30	100	<LOQ	Paclobutrazol	6.5000E-2	30	100	<LOQ
Chlormequat Chloride	1.0800E-1	10	3000	<LOQ	Pentachloronitrobenzene	1.3200E+0	10	200	<LOQ
Chlorpyrifos	3.5000E-2	30	100	<LOQ	Permethrin	3.4300E-1	30	1000	<LOQ
Clofentezine	1.1900E-1	30	500	<LOQ	Phosmet	8.2000E-2	30	200	<LOQ
Coumaphos	3.7700E+0	48	100	<LOQ	Piperonylbutoxide	2.9000E-2	30	3000	<LOQ
Cyfluthrin	3.1100E+0	30	1000	<LOQ	Prallethrin	7.9800E-1	30	400	<LOQ
Cypermethrin	1.4490E+0	30	1000	<LOQ	Propiconazole	7.0000E-2	30	1000	<LOQ
Daminozide	8.8500E-1	30	100	<LOQ	Propoxur	4.6000E-2	30	100	<LOQ
Diazinon	4.4000E-2	30	200	<LOQ	Pyrethrins	2.3593E+1	30	1000	<LOQ
Dichlorvos	2.1820E+0	30	100	<LOQ	Pyridaben	3.2000E-2	30	3000	<LOQ
Dimethoate	2.1000E-2	30	100	<LOQ	Spinetoram	8.0000E-2	10	3000	<LOQ
Dimethomorph	5.8300E+0	48	3000	<LOQ	Spinosad	8.8000E-2	30	3000	<LOQ
Ethoprophos	3.6000E-1	30	100	<LOQ	Spiromesifen	2.6100E-1	30	3000	<LOQ
Etofenprox	1.1600E-1	30	100	<LOQ	Spirotetramat	8.9000E-2	30	3000	<LOQ
Etoxazole	9.5000E-2	30	1500	<LOQ	Spiroxamine	1.3100E-1	30	100	<LOQ
Fenhexamid	5.1000E-1	10	3000	<LOQ	Tebuconazole	6.7000E-2	30	1000	<LOQ
Fenoxycarb	1.0700E-1	30	100	<LOQ	Thiacloprid	6.4000E-2	30	100	<LOQ
Fenpyroximate	1.3800E-1	30	2000	<LOQ	Thiamethoxam	5.0000E-2	30	1000	<LOQ
Fipronil	1.0700E-1	30	100	<LOQ	Trifloxystrobin	3.7000E-2	30	3000	<LOQ
Flonicamid	5.1700E-1	30	2000	<LOQ					

Aixia Sun Lab Director/Principal Scientist
D.H.Sc., M.Sc., B.Sc., MT (AAB)

Definitions are found on page 1

This report shall not be reproduced, without written approval, from ACS Laboratory. The results of this report relate only to the material or product analyzed. Test results are confidential unless explicitly waived otherwise. ACS Laboratory is accredited to the ISO/IEC 17025:2017 Standard.

ACCREDITED CERT #6786.01

DEA REGISTERED LABORATORY #RA0571996

AHCA

QA By: 1042 on 2023-08-13 14:04:39 V1
