# Supplementary material for: Development and Characterization of Cannabidiol Gummy Using 3D Printing
Source: Gels. 2025 Mar 8;11(3):189. doi: 10.3390/gels11030189 (PMC11941846; doi:10.3390/gels11030189)
Supplement: Supplementary file 1 [file gels-11-00189-s001.zip › gels-3445528-supplementary.pdf]

gels

Article

Development and Characterization of Cannabidiol Gummy Using 3D Printing

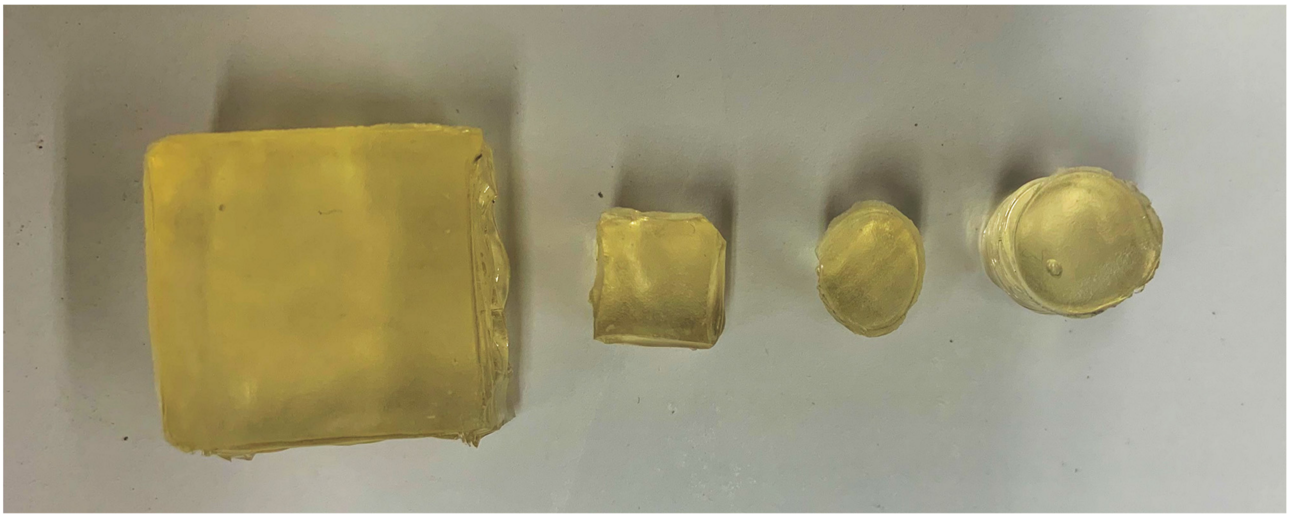

Figure S1. CBD gummies with different shapes and sizes printed using 3D printer.

Table S1: Different batches gummies with various shapes and sizes showing so significant differences in their weight, size, and shape.

Gummy Batch	Shape	Size	Weight (mg)
B-1	Rectangular cuboid	20 × 10 mm	5340.60 ± 298.88
B-2	Cube	10 mm each side	762.00 ± 39.92
B-3	Ellipsoid	10 × 4 × 10 mm	456.60 ± 15.56
B-4	Cylindrical	10 ×10 mm	657.60 ± 34.72
